# Processing of nerve biopsies: A practical guide for neuropathologists 

**DOI:** 10.5414/NP300468

**Published:** 2011-12-27

**Authors:** Joachim Weis, Sebastian Brandner, Martin Lammens, Claudia Sommer, Jean-Michel Vallat

**Affiliations:** 1 Institute of Neuropathology, Medical Faculty, RWTH Aachen University, Aachen, Germany,; 2 Division of Neuropathology, National Hospital for Neurology and Neurosurgery, and Department of Neurodegenerative Disease, UCL Institute of Neurology, London, UK,; 3 Departments of Pathology and Neurology, Radboud UMC Nijmegen, Nijmegen, The Netherlands,; 4 Department of Neurology, University Hospital of Würzburg, Würzburg, Germany,; 5 Neurology Department, National Reference Center: “Rare peripheral neuropathies”, University Hospital, Limoges, France

**Keywords:** nerve biopsy

## Abstract

Nerve biopsy is a valuable tool in the diagnostic work-up of peripheral neuropathies. Currently, major indications include interstitial pathologies such as suspected vasculitis and amyloidosis, atypical cases of inflammatory neuropathy and the differential diagnosis of hereditary neuropathies that cannot be specified otherwise. However, surgical removal of a piece of nerve causes a sensory deficit and – in some cases – chronic pain. Therefore, a nerve biopsy is usually performed only when other clinical, laboratory and electrophysiological methods have failed to clarify the cause of disease. The neuropathological work-up should include at least paraffin and resin semithin histology using a panel of conventional and immunohistochemical stains. Cryostat section staining, teased fiber preparations, electron microscopy and molecular genetic analyses are potentially useful additional methods in a subset of cases. Being performed, processed and read by experienced physicians and technicians nerve biopsies can provide important information relevant for clinical management.

## Introduction 

With a prevalence of 1 : 200 [1], peripheral neuropathies (PNP) encompass one of the largest disease groups among the neurological disorders. The causes of PNP are manifold, including metabolic, inflammatory, degenerative, toxic, hereditary, vascular, malnutritive, paraneoplastic and other processes. Even though clinical history and examination combined with electrophysiological and laboratory methods often uncover the cause of PNP, a substantial number of cases remain unsolved and stay without definite diagnosis after careful application of these methods. In such situations, nerve biopsies have been a method of choice for decades to classify PNPs and to find clues to uncover their etiology. 

This short review is intended to provide a guide for practicing neuropathologists how to approach nerve biopsies. It is based on recently published guidelines [2, 3], contemporary reviews [4, 5, 6], textbooks including [7, 8, 9, 10, 11], and the personal experience of its authors. 

## Indications 

Defining indications for a nerve biopsy is beyond the scope of this neuropathological review. In our experience, and based on the results published by others, nerve biopsy can be diagnostically helpful and valuable as part of the therapeutic decision making process, especially if inflammation or other interstitial pathology such as vasculitis, granulomatous inflammation, amyloidosis or atypical CIDP is suspected [2, 3, 12]. In the usual clinical setting, the major rationale to perform a nerve biopsy is to gain information about therapeutic options when inflammatory neuropathy is considered. For example, immunosuppressive drugs can present a risk due to their side effects, or intravenous immunoglobulins are expensive. Nerve biopsies have also been found to be useful to detect pathological immunoglobulin deposits [1]. In addition, they can provide guidance in the differential diagnosis of hereditary neuropathies with atypical presentation or ambiguous genetic testing results, identify pathological features in the context of a suspected genetic condition, or detect an inflammatory component in hereditary neuropathies. In many cases, combined etiologies are uncovered by nerve biopsy analysis, including microangiopathic/diabetic and inflammatory or hereditary and inflammatory, which is also helpful for clinical management. 

Prior to nerve biopsy, a complete clinical, electrophysiological and laboratory workup is mandatory. Nerve biopsies should only be performed by medical professionals experienced in the procedure, and appropriate histological processing must be available. The potential benefits for the patient must outweigh the discomfort of the biopsy procedure itself and the side effects including the sensory deficit and in some cases chronic pain. 

## Sites 

The specimen should be obtained from an affected nerve. Most neuropathies show distal accentuation. The sural nerve is purely sensory in more than 90% of patients and contains only few motor fibers in the remaining patients [13]. Moreover, it is easily accessible to surgery and therefore most frequently chosen for biopsy. It usually contains between 5 and 10 nerve fascicles. In a large autopsy study, 3,300 – 8,000 myelinated and 10,500 – 45,500 unmyelinated nerve fibers were found in subjects without history of disease or ingestion of drugs known to affect peripheral nerve [14]. However, it should be pointed out that it may not always be advisable to select the nerve that is most significantly affected in nerve conduction studies, as a terminally depleted nerve will be less informative than a nerve with a population of residual fibers. 

Some groups published larger series of biopsies of other nerves including the superficial peroneal nerve as fragments from the adjacent peroneus brevis muscle may be taken during the same surgical procedure (see below: vascular changes). The superficial radial nerve may be chosen when symptoms predominate in the upper limbs. Obturator nerve biopsy is considered to be quite useful to differentiate motor neuropathies and lower motor neuron diseases [15, 16]. 

In selected cases with suspected focal lesions, biopsies of larger, mixed sensory and motor nerves guided by MR imaging and/or sonography can be performed to detect and classify inflammation (focal neuritis), neoplasias (nerve sheath tumors, perineuriomas, neurolymphomatosis and others) and hereditary hypertrophic neuropathies. 

## Surgical procedures 

The nerve segment should be excised inflicting minimal mechanical injury. Squeezing or stretching the nerve should be strictly avoided and excessive removal of fat or connective tissue should not be attempted. Nerve fibers are very sensitive to mechanical injury (Figure 6A, B). The proximal nerve cut should be performed first, as cutting the nerve often causes acute pain even under local anesthesia. Instead, if no pain is reported at all when the “nerve” is transsected, a blood vessel may have been mistaken for a nerve. Do not place the proximal stump of the dissected nerve immediately under the skin or even fix it to the skin by a suture, as this increases the likelihood of formation of a painful, irritable neuroma. Biopsy of the medial sural nerve and fixation of the proximal stump in the gastrocnemic muscle has been suggested to reduce side effects such as persistent pain and neuroma formation; in addition, this more proximal sural nerve biopsy is more easily combined with a gastrocnemic muscle biopsy [17]. 

Biopsy of just a few fascicles does not cause fewer complications or less sensory deficit than transecting the entire nerve, and a fascicular biopsy provides less tissue for analysis. This is especially important if focally accentuated lesions including inflammatory processes are looked for. Such foci are less likely to be detected if just a portion of the nerve is examined. Therefore, biopsy of the complete cross section of the nerve is recommended [18]. 

The recommended length of the biopsied nerve segment is 5 cm. Removal of a shorter segment will be diagnostically less useful or can hamper a proper analysis, but will leave an identical sensory deficit. 

## Neuropathological work-up 

Immediately after removal the proximal 1.5 – 2 cm piece of this segment should be frozen without fixation in isopentane cooled by liquid nitrogen. The distal 1.5 – 2 cm piece should be fixed in buffered 10% formalin. The central piece should be fixed in 3.9% glutaraldehyde (e.g., Merck No. 104239) in 0.4 molar phosphate buffer, pH 7,6 (for 1,000 ml: 7,176 g NaH_2_PO_4_ × H_2_O, Merck no. 106346, and 49.4 g Na_2_HPO_4_, Merck No. 106586) and thereafter incubated with 1% osmium tetraoxide for epoxy resin embedding and subsequent semithin cross and longitudinal sectioning, electron microscopy and teased fiber preparation where indicated. Dividing the excised nerve segment this way is recommended because cryostat and paraffin section morphology is less sensitive to cutting artifacts [10]. 

Formalin and glutaraldehyde fixation of nerve segments is essential, freezing an unfixed segment is optional, but recommended. Frozen sections can be cut and stained immediately after biopsy for rapid initial screening, e.g., to confirm suspected vasculitis. In addition, cryostat sections can be used for immunohistochemistry in the case of antigens that are not detectable after paraffin embedding or to perform immunofluorescence (see below). Finally, the frozen nerve segment may also be a good source for RNA and protein studies and, if a blood sample is not available, for DNA for molecular pathology, molecular genetics and biochemistry. Frozen material should be stored in a –80 °C freezer for studies which might be of therapeutic relevance, e.g., for the definition of subgroups with differential response to specific treatments that might come up in the future. 

### Cut-up and sectioning

The formalin-fixed nerve segment is dissected in 2 – 4 pieces which are arranged transversely and longitudinally in a paraffin block to cut sections of 3 – 4 µm thickness. Serial sectioning of 3 – 4 levels or alternatively 30 consecutive sections is recommended if an inflammatory, especially a vasculitic neuropathy is suspected, but has not been detected in the initial sections. It is also recommended to archive unused unstained sections, especially if only very little tissue is left in the paraffin block, as they may become useful for additional stains. 

### Tinctorial stains

The recommended stains include H&E, Congo red, and Turnbull or Pearl’s (Prussian blue) for iron depositions. Thioflavin S or T for more sensitive amyloid detection (requires fluorescence microscope), Gomori trichrome, Ladewig, elastica van Gieson, and myelin stains such as luxol fast blue are optional. Immunohistochemical stains for myelin and axonal (neurofilament) proteins provide an alternative to conventional myelin and axon stains. However, semithin section resin histology (see below) is always preferable over paraffin section histology and immunohistochemistry for myelin and axon staining. 

### Immunohistochemistry

Immunohistochemistry on paraffin sections should encompass at least LCA (CD45RO, pan leukocyte), CD3 (pan-T cells), and CD8 (cytotoxic T cells). Further optional stains include CD4 (T helper) and CD20 (B cells), smooth muscle antigen (SMA) to detect vessel wall alterations due to vasculitis as well as neurofilament and PGP9.5 to stain axons, immunoglobulin and light chain antibodies, transthyretin, EMA (for cells with perineurial differentiation) and S100 (Schwann cells). Myelin Basic Protein (e.g., antibody SMI94) is more specific for myelin sheaths than tinctorial myelin stains and will give a significantly better signal to noise ratio, in particular if only very few, small myelinated fibers are preserved. Immunofluorescence of frozen sections is an alternative to conventional immunohistochemistry of paraffin sections mainly to detect deposits of abnormal immunoglobulins either in the endoneurial space or in myelin sheaths. The same technique can be useful to immunolabel amyloid deposits formed by light chain or transthyretin. The specific types of endoneurial lymphocyte infiltration may be also confirmed by immunohistochemical examination of frozen sections. Most laboratories, however, prefer paraffin section immunohistochemistry for the latter purpose. 

### Semithin resin cross and longitudinal sections

Semithin resin cross and longitudinal sections are essential, because they provide a much more comprehensive and detailed picture with higher resolution and morphological accuracy of the relevant structures including axons and myelin sheaths than paraffin sections. Toluidine blue and methylene blue-azure II give better contrast and allow for the detection of metachromatic material, but fade more easily over the years than the brown paraphenylendiamine stain. The longitudinal semithin sections facilitate analysis of the nodes of Ranvier and of adjacent internodes. 

### Teased fiber preparations

Teased fiber preparations (Figure 2) are laborious and can be hard to obtain considering the restricted number of technical personnel in most laboratories. However, if done adequately on a sufficient number of nerve fibers (~100) they provide valuable information on the extent and progression of fiber degeneration, and may demonstrate regeneration as well as axonal atrophy, axonal swellings, de- and remyelination and tomacula [3, 7]. Longitudinal semithin sections may provide a good although not perfect alternative to teased fiber preparations. 

### Transmission electron microscopy (TEM)

**Figure 1.**
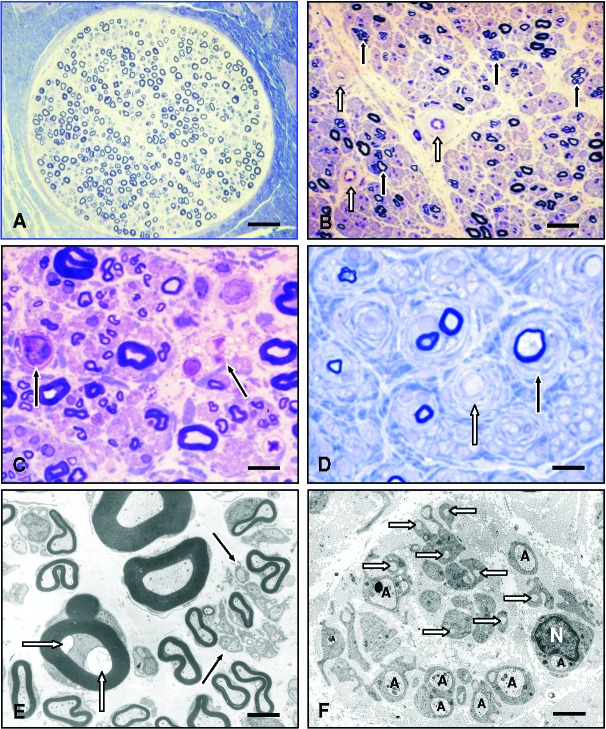
Normal and basic pathological nerve biopsy findings. A: Sural nerve fascicle of a 65-year-old patient showing normal density and distribution of large and small myelinated nerve fibers. Semithin section, toluidine blue. Scale bar = 80 µm. B: Diabetic neuropathy: Axonal neuropathy pattern with reduced myelinated nerve fiber density and clusters of regenerating nerve fibers (black arrows). Marked thickening of endoneurial blood vessel walls (white arrows) due to basal lamina reduplication and widening. Semithin section, toluidine blue. Scale bar = 50 µm. C: Rapidly progressive axonal neuropathy associated with acute nerve fiber breakdown. Arrows: macrophages containing myelin debris. Semithin section, toluidine blue. Scale bar = 15 µm. D: Demyelinating neuropathy (CMT1A). Schwann cell processes are forming onion bulbs around a myelinated (black arrow) and a non-myelinated axon. Semithin section, toluidine blue. Scale bar = 15 µm. E: Normal ultrastructural appearance of large and small myeunated, and unmyelinated (black arrows) nerve fibers. White arrows: retraction of the axon from the inner mesaxon, a frequent artefact. EM. Scale bar = 3 µm. F: Loss of unmyelinated axons (A) associated with numerous Schwann cell processes ensheathing bundles of collagen fibers, so called empty collagen pockets. N = Schwann cell nucleus. EM. Scale bar = 2 µm.

Transmission electron microscopy (TEM) of ultrathin sections contrast-enhanced with uranyl acetate and lead citrate are important for the detection of changes of unmyelinated fibers including denervated Remak bundles, so-called collagen pockets (non-myelinating Schwann cells ensheathing bundles of collagen fibers instead of axons) (Figure 1F), and abnormal processes of non-myelinating Schwann cells, as found in CMT4C (Figure 5D). Such changes cannot be examined with any other method. In addition, TEM may demonstrate other important features which can be hard to detect by light microscopy such as uncompacted/decompacted (Figure 4C) or focally folded myelin (Figure 5C), macrophage-mediated demyelination, subtle immunoglobulin and amyloid deposits (Figure 4D) or pathological inclusions as found in metachromatic leukodystrophy, adrenomyeloneuropathy or amiodarone intoxication. 

### Morphometry

Morphometry is used to precisely determine the extent of nerve fiber loss and of axonal vs. myelin sheath degeneration [19]. It is not in regular use in routine diagnostics, but often necessary in scientific studies on human biopsies and animal models. 

Results of nerve biopsies should be discussed with the clinicians in the context of the clinical, laboratory and electrophysiological findings. Difficult cases can be referred to a reference center, e.g., the Reference Center for Neuromuscular Diseases of the German Society of Neuropathology and Neuroanatomy (DGNN; http://www.neuromuskulaeres-referenzzentrum.dgnn.rwth-aachen.de/index.html; head: J. Weis, Aachen, Germany), the Neuromuscular Center at UCL Institute of Neurology (S. Brandner) or the National Reference Center: “Rare peripheral neuropathies”, University Hospital, Limoges, France (J.-M. Vallat), for a second opinion and further study. 

Many laboratories keep sections, tissues and files of nerve biopsy cases indefinitely. Quite often, this material can be helpful after years and even decades, for instance for the patient him/herself or for his/her relatives in the context of genetic analyses as well as for scientific studies. 

Combined nerve and muscle biopsies can be obtained by the same skin incision, but in separate pieces. The chance to detect inflammatory changes, in particular vasculitis, is significantly higher in a combined biopsy compared to either nerve or muscle biopsy alone [20]. In addition, other disorders that can affect both nerve and muscle such as amyloidosis or certain hereditary diseases including neuropathy and myopathy due to dynamin 2 mutation [21] are more likely to be detected. Finally, in motor neuron diseases a discrepancy between the severe, often rapidly progressive neurogenic muscular atrophy and the mildly affected or even normal-appearing sural nerve is detected. 

## Skin biopsy 

Biopsies of the skin are used to examine the various nerve fiber populations of the epidermis and the dermis, in particular the small, unmyelinated epidermal nerve fibers. 3 – 4 mm punch biopsies are obtained usually at the standard location 10 cm proximal to the lateral malleolus and at the proximal thigh and fixed in Zamboni solution or buffered paraformaldehyde. 40 – 50 µm cryostat sections are stained immunohistochemically using a PGP9.5 antibody to examine epidermal nerve fiber density and morphology, density of the subepidermal plexus and sweat gland innervation (Figure 6C, D, E). The most frequent indication for neurological skin biopsy is suspected small fiber neuropathy. This disorder is hard to detect in sural nerve biopsies, because, even though this nerve is located rather distally, it does not contain the most distal nerve fiber endings. Moreover, analysis of the unmyelinated nerve fiber population in the sural nerve requires electron microscopy, which is not available in every lab. Furthermore, inclusion of a proximal biopsy gives valuable information on whether the neuropathy is length-dependent or not, and immunohistochemistry for inflammatory cells may be used as an additional tool to detect vasculitis [22]. Finally, skin punch biopsy is – of course – less invasive than sural nerve biopsy and can be repeated for follow-up studies, e.g., in pharmaceutical or other therapeutic trials. For the analysis, it is important to strictly adhere to internationally agreed counting methods and compare the results with age matched controls, as there is considerable age- and sex-matched variation [23]. 

## Pitfalls

In up to 4% of cases, a blood vessel, usually a vein, is mistaken for the sural nerve and biopsied. More frequently, the biopsied nerve segment is mechanically damaged due to inadequate handling of the biopsy during removal (see above). Other frequent handling artefacts include shrinkage due to hyperosmolar fixative or freezing the nerve prior to or after fixation. The latter mistake has to be avoided especially in the context of combined muscle/nerve biopsies. Do not place any fixed tissue, neither muscle nor nerve, into the styrofoam box together with the fresh frozen tissue. Do not tape the tubes with the fixed tissue to the styrofoam box containing dry ice such that leaking cold temperature can freeze the tubes.
As mentioned above, glutaraldehyde fixation is required for plastic embedding and subsequent semithin and ultrathin sections. If the entire nerve biopsy is immediately placed in formalin or in a mixture of formalin and glutaraldehyde, fixation will be suboptimal for semithin sections and electron microscopy. In case of fixation with a mixture of formalin and glutaraldehyde, the formalin more quickly penetrates the tissue which means that essentially a formalin fixation with glutaraldehyde post-fixation takes place, resulting in severe artefacts such as myelin splitting. In addition, glutaraldehyde-fixed tissue is less suitable for most immunohistochemical stains. This is due to the stronger denaturing properties of glutaraldehyde resulting in antigen masking.
Keep in mind that myelin sheaths do not reach their full thickness until age 15 [24] and that nerve fiber degeneration and regeneration as well as de- and remyelination can occur more frequently in subjects above the age of 60 years [14].
Do not mistake Renaut bodies (Figure 6C), a sequel of chronic compression injury (see below), for nerve infarcts.
In most cases, when nerve biopsy material is forwarded to a more specialized laboratory it is usually sufficient to send just glutaraldehyde and formalin-fixed tissue initially. This can be sent by normal surface mail at normal temperature. Express delivery or cooling is not necessary.

## What to look for in a nerve biopsy 

Features to be described in any nerve biopsy report are listed in Table 1. 

### Vascular changes

Microangiopathy is among the most frequent alterations observed in nerve biopsies. Typically, the endothelia are hyperplastic and the basal laminae of endo- and epineurial blood vessels are thickened and reduplicated (Figure 1B). Such changes are often found in the context of diabetic neuropathy [25, 26], but can also be found in non-diabetic patients, possibly preceding manifest diabetes mellitus. Atherosclerosis of small epineurial arteries is present quite frequently and typically is more prevalent in biopsies of elderly patients or in patients with a history of hypertension, whereas media calcification (Mönckeberg’s sclerosis) is a rare finding of unclear significance. The granular osmiophilic deposits of cerebral autosomal dominant angiopathy with subcortical infarcts and leukoencephalopathy (CADASIL) can be detected in sural nerve biopsies by EM [27]; however, skin biopsy is usually sufficient to detect these changes. 

### Inflammatory alterations

Guillain-Barré syndrome (GBS) is characterized by multifocal and randomly distributed juxtanodal areas of demyelination accompanied by focally accentuated lymphocytic infiltration of the endoneurium (Figure 3A) and endoneurial edema. Sural nerve biopsy is rarely performed in such patients, because the diagnosis is made with sufficient certainty in most cases based on clinical, electrophysiological and CSF findings in most cases. Chronic neuritis (chronic inflammatory demyelinating neuropathy, CIDP, or chronic inflammatory axonal neuropathy, CIAP) is more often diagnosed by nerve biopsy, especially in atypical cases [28]. In chronic neuritis, endoneurial edema, endoneurial macrophage clusters [29] as well as increased numbers of CD8-immunoreactive cytotoxic T lymphocytes (Figure 3B, C) are found [30]. However, these changes are not specific for chronic neuritis, as they can, for example, also be present in peripheral nerve vasculitis. The detection of active macrophage-mediated demyelination pathognomonic for an acquired demyelinating neuropathy often requires electron microscopy. 

Suspected vasculitis, often in the context of connective tissue disease, multiple mononeuropathy or progressive axonal neuropathy is the most frequent indication for nerve biopsy. Involvement of the PNS in systemic vasculitis is infrequent, but selective vasculitis of peripheral nerve blood vessels can occur. Non-systemic vasculitic neuropathy [31] is a treatable PNP that, up to date, can only be diagnosed by nerve biopsy, although inflammatory infiltrates in muscle and skin may support the diagnosis. Vasculitis is defined by inflammatory infiltrates leading to vessel wall necrosis (Figure 3D). Vessel wall necrosis may be accompanied by hemorrhage into the vessel wall and/or thrombosis. It often follows the pattern of unspecific lymphocytic angiitis, which is frequently found in systemic connective tissue diseases such as rheumatoid arthritis. A minor reactive lymphocytic infiltration of the wall of an epineurial blood vessel or of the perivascular tissue does not qualify for the diagnosis of a vasculitis. However, detection of such a minor infiltration should prompt further analysis of the biopsy by serial sections stained immunohistochemically (LCA, CD3, CD8, CD68). Serial sections should also be analyzed if inflammatory infiltrates are absent in the initial sections of a biopsy if the clinical data are suggestive of a PNS vasculitis. 

If perivascular lymphocytic cuffing or infiltration or moderate infiltration of blood vessel walls is accompanied only by active, focally accentuated or fascicular nerve fiber loss, but no vessel wall necrosis, the terms “non-necrotizing vasculitis” or “probable vasculitis” can be used [32, 33]. Organized vascular occlusion with recanalization (Figure 3E), organized hemorrhage detected by iron stains (Figure 3F), and focal proliferation of small vessels are additional signs of non-florid vasculitis. 

In some cases, peripheral nerve vasculitis can be classified as panarteritis nodosa, characterized by florid infiltration by lymphocytes and granulocytes affecting small arteries, often accompanied by fibrinoid necrosis (Figure 3D), or microscopic polyangiitis, which also targets arterioles and venules. Peripheral nerves can be involved in Churg-Strauss syndrome (CSS) and other special rheumatological disorders such as Sjögren’s syndrome. As mentioned above, combined nerve and muscle biopsy may improve the diagnostic yield in the detection of definite vasculitis [20]. 

### Sarcoidosis

The typical non-caseating granulomatous lesions of sarcoidosis are usually found in the epineurium. Chronic necrotizing vasculitis in the absence of granulomas can be present in other cases of sarcoidosis. The inflammatory infiltration is mainly associated with axonal neuropathy; only rarely a predominant demyelinating pattern is seen [34]. Again, combining nerve biopsy with muscle biopsy may increase the chance of detecting the pathognomonic lesions [34]. Small fiber neuropathy is a frequent complication of sarcoidosis and can be diagnosed by skin biopsy [35]. 

### Infectious disorders

Borreliosis also leads to lymphocytic infiltration of epineurial blood vessel walls associated with perineurial thickening and fibrosis and axonal neuropathy. A characteristic pattern of perineurial TNF-α, C5b-9, and ICAM-1 expression has been found in sural nerve biopsies of borreliosis patients [36]. 

HIV infection can cause a non-inflammatory, mostly sensory neuropathy [37] which can also be caused by antiretroviral therapy. In addition, GBS or chronic neuritis/CIDP type neuropathy as well as chronic necrotizing PNS vasculitis leading to a multiplex mononeuropathy can occur in conjunction with HIV infection [38]. Other viral infection, especially with hepatitis C, can be associated with necrotizing PNS vasculitis [32]. 

Leprosy is one of the most frequent neuromuscular diseases worldwide with a high prevalence, for example, in Brazil or India. Histological diagnosis is important, which is often achieved by skin biopsy, but frequently also requires nerve biopsy. Lepromatous leprosy is characterized by chronic inflammatory infiltrates with masses of acid-fast bacilli in histiocytes (Figure 4A) which can occur in any compartment of the nerve; in contrast, bacilli are only rarely detected in the granulomatous lesions of tuberculoid leprosy (Figure 4B) [5]. Painful chronic inflammatory neuropathy associated with histologically detectable acid-fast bacilli has been observed in leprosy patients even after multidrug therapy [39]. 

### Neuropathies linked to a monoclonal gammopathy

A link between the PNP and the monoclonal dysglobulinemia has to be established and a coincidental association has to be eliminated. Different mechanisms can be detected by nerve biopsy and an exact mechanism has to be established to discuss a specific treatment. Otherwise, if such patients are treated, neurotoxic lesions which may be induced by cytostatic drugs (see below) also have to be excluded. 

The nerve lesions may be due to endoneurial deposits of immunoglobulins. IgM with anti-MAG (myelin associated glycoprotein) activity usually induces deposits in the myelin sheaths which can be detected specifically by immunofluorescence and which correspond to characteristic widenings of the space between myelin lamellae at the ultrastructural level [40] (Figure 4C). Actually, nerve biopsy is now rarely indicated if anti-MAG antibodies are significantly elevated in the serum of the patient. If there is an IgG or IgA monoclonal dysglobulinemia, direct immunofluorescence may rarely demonstrate annular bindings to many myelinated fibers, corresponding to IgA or IgG deposits. Immunoglobulin deposits of any kind (IgG, IgA or IgM) in the interstitial tissue can only be demonstrated by nerve biopsy [41]. In practice, it may be difficult to identify such deposits using paraffin sections, as they are usually scarce. Moreover, immunocytochemistry often fails to demonstrate immunoglobulin deposits in paraffin-embedded samples. If they are big enough, they can be seen by immunofluorescence on frozen sections; if they are small, electron microscopy (Figure 4D) has to be used. 

In POEMS syndrome, specific immunocytochemistry study of myelinated fibers and of the endoneurium may be negative but electron microscopy can disclose uncompacted myelin lamellae (UML) which are characteristic features of this condition and may be visible in about 10% of the persisting myelinated fibers [42]. 

### Amyloidoses

Both primary (AL) amyloidosis due to immunoglobulin light chain deposition and familial ATTR amyloidosis due to transthyretin mutations often affect the peripheral nerve. For the histological diagnosis, the Congo red stain can be used as a screening method (Figure 4E). However, Congo red is much less sensitive than the Thioflavin S (Figure 4F) or T fluorescent stains. Amyloid deposits can also be detected in toluidine-blue stained semithin sections. They are often focally distributed; therefore, serial sections of multiple blocks should be searched for deposits in case amyloid neuropathy is suspected. Suspicion can be raised both based on clinical grounds and on the typical histological pattern of severe nerve fiber loss with predominant involvement of the small myelinated as well as unmyelinated nerve fibers. In some cases, EM can be helpful to detect or verify amyloid deposition. 

Immunohistochemistry with antibodies against transthyretin, amyloid A component, immunoglobulin and light chain antibodies can be used to type amyloid deposits. Recently, luminescent-conjugated polymer spectroscopy has been introduced as a new method to characterize amyloid deposits in histological sections [43]. 

### Toxic neuropathies

Alcoholic neuropathy is characterized by an axonal loss which predominantly affects small nerve fibers; in contrast, the axonal neuropathy due to thiamine deficiency has been reported to affect mainly large fibers [44]. In chronic alcoholic neuropathy clusters of regenerating nerve fibers may be found in large numbers. 

Cytostatic drugs frequently lead to severe neuropathy. In fact, neuropathy is often a dose-limiting side effect. Taxol, vincristine and cisplatin induce predominantly axonal neuropathy. Other drugs, such as amiodarone and chloroquine, cause predominantly demyelinating neuropathy with characteristic inclusions which can be detected by EM. 

### Neuropathies associated with neoplasias

**Figure 2.**
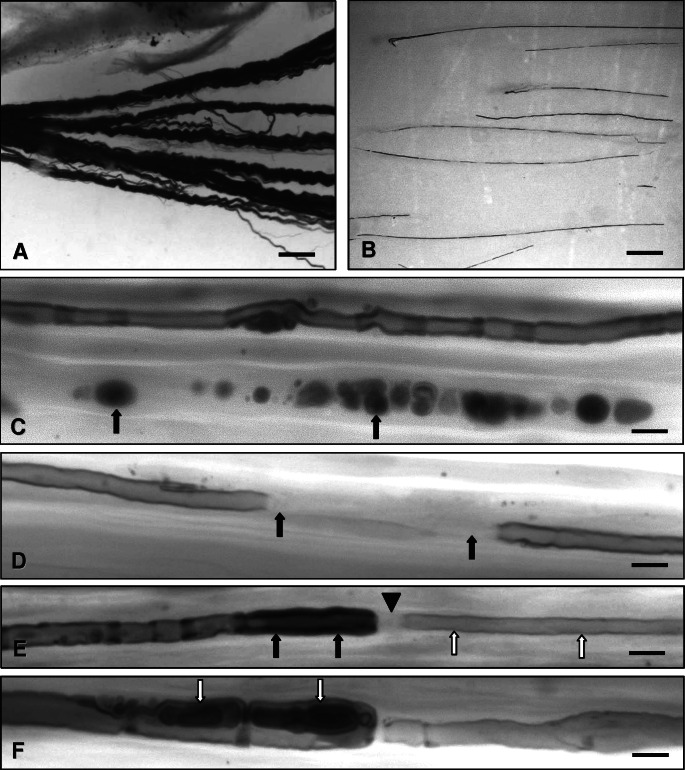
Teased fiber studies. A: Separation of groups of nerve fibers at the start of the teasing procedure. Scale bar = 100 µm. B: Individual teased fibers. Scale bar = 100 µm. C: Myelin breakdown (myelin spheres or ovoids; arrows) indicating nerve fiber breakdown in axonal neuropathy. Scale bar = 15 µm. D: Demyelinated nerve fiber segment (arrows). Scale bar = 15 µm. E: Hypermyelinated paranodal segment (black arrows), thinly remyelinated internode (white arrows) and enlarged node of Ranvier (arrowhead) in demyelinating neuropathy. Scale bar = 15 µm. F: Paranodal dysmyelination (focally folded myelin, FFM; arrows) in demyelinating neuropathy. Scale bar = 10 µm.

Paraneoplastic neuropathy in patients with solid tumours such as small-cell lung cancer is often associated with autoantibodies including anti-Hu or anti-CV2 and leads to rapid nerve fiber breakdown with numerous myelin ovoids (Figure 2C). Bands of Büngner and endoneurial macrophages are frequently encountered. Clusters of regenerating nerve fibers are rare, in line with the severe type of injury due to lymphocytic infiltration of the dorsal root ganglia and multifocal microvasculitic infiltration of the nerves [45]. 

Direct infiltration of peripheral nerves by carcinomas is a common feature of advanced tumor progression; however, it is only rarely encountered in nerve biopsies performed in the context of peripheral neuropathy. On the other hand, diffuse infiltration of peripheral nerves by malignant lymphomas is rather common and may involve the sural nerve. Spread of the lymphoma to the peripheral nerves (neurolymphomatosis) may even occur early in the course of the disease, thus mimicking neuritis [46]. 

Neoplasms of the peripheral nerves such as schwannomas, neurofibromas and malignant peripheral nerve sheath tumors (MPNSTs) often occur as solitary lesions not involving the sural nerve. However, hereditary tumor syndromes, in particular neurofibromatosis (NF) 1 and 2, may affect the PNS in a more diffuse manner: NF1 with subcutaneous and large root diffuse neurofibromas can be associated with peripheral neuropathy [47], whereas NF2 is characterized by disseminated abnormal endoneurial Schwann cell proliferations, so called tumorlets [48]. 

### Hereditary neuropathies

Today, a definite diagnosis of straightforward hereditary neuropathy cases is often established by molecular genetic testing. However, nerve biopsies are still performed if hereditary NP is ascertained by clinical and family history, but analyses of the most common genes did not yield a diagnosis. In such cases, the nerve biopsy findings can help to narrow down the potential disease gene. Moreover, morphological patterns suggestive of a hereditary neuropathy are often found by chance in nerve biopsies from patients with seemingly sporadic neuropathy. 

Mutations in more than 50 genes have been identified so far that lead to hereditary sensory and motor and hereditary sensory and autonomic neuropathy (HSMN and HSAN, respectively). A comprehensive description of the vast landscape of alterations found in nerve biopsies in these disorders is beyond the scope of the present review. Briefly, special features of predominantly demyelinating hereditary neuropathies include focal myelin thickenings (tomacula (Figure 5B) most frequently found in hereditary neuropathy with liability to pressure palsy, HNPP, due to PMP22 gene duplication), focally folded myelin (FFM (Figures 2F, 5C) mainly in recessive demyelinating CMTs such as CMT4B2 due to frabin/SBF2 mutation [49]), abnormal myelin compaction [50], and basal lamina onion bulbs as well as abnormal processes of Schwann cells of unmyelinated nerve fibers (Figure 5D) and node of Ranvier widening as seen in CMT4C [51]. Onion bulb formations consisting of surplus Schwann cell processes and Schwann cell basal laminae are a characteristic feature of chronic hereditary demyelinating neuropathy, such as CMT1A and B (Figure 1D), but can also be found in CIDP. 

The genetic basis of most hereditary axonal neuropathies remains to be defined. Therefore, matching typical axonal alterations with certain mutations is still difficult. At present, mitofusin 2 mutations appear to be a relatively frequent cause of axonal CMT. In sural nerve biopsies of such cases prominent intraaxonal aggregates of organelles including enlarged mitochondria that show an abnormal structure are detected by EM [52]. Mutations in the neurofilament light chain (NEFL) gene lead to ultrastructural alterations of the axonal cytoskeleton [53]. 

NEFL mutations can even cause prominent swellings of axons, which are visible by light microscopy [53] and similar to the large focal axonal distensions found in giant axon neuropathy (Figure 5A) due to gigaxonin mutation [54, 55]. Giant axonal neuropathy affects both the PNS and the CNS. Another disorder that involves both PNS and CNS nerve fibers (as well as skeletal muscle) is polyglucosan body disease [56, 57, 58]. It is caused by mutations in the glycogen branching enzyme 1 (GBE1). In peripheral nerves, polyglucosan bodies are typically found in axons. Solitary intraaxonal polyglucosan bodies can be detected as an unspecific, apparently age-related finding in sural nerve biopsies of patients with various types of neuropathy [59]. 

The PNS is frequently involved in various lipidoses [4]. For instance, characteristic inclusions are observed in Fabry’s disease (concentric lamellar inclusions in perineurial and endothelial cells as well as Schwann cells [60], metachromatic leukodystrophy (prismatic and lamellar, herringbone-like inclusions in Schwann cells and macrophages [61]), and adrenoleukodystrophy (lamellar inclusions and trilaminar leaflets [62]). Nowadays, a specific gene mutation has been linked to many of these disorders; therefore molecular biology may be considered as a diagnostic procedure preceding nerve biopsy. 

### Compression injury

Chronic nerve compression leads to fibrosis of the epi-, peri- and endoneurium, endoneurial edema, de- and remyelination and axonal loss. Endoneurial deposits of mucoid substance are frequently encountered. Renaut bodies (Figure 6C) are composed of sparse, concentrically arranged, elongated fibroblast-like cells surrounded by ample mucoid extracellular matrix that contains precursors of elastic fibers [63]. These structures are usually found in a subperineurial location and are frequent at sites of chronic nerve compression, i.e., in the median nerve within the carpal tunnel. They are often present in the sural nerve and should not be mistaken for organized nerve infarcts. 

### Combinations of different causes of neuropathy

In the clinical context, it can be particularly helpful to find evidence for two or more causes of peripheral neuropathy in a given nerve biopsy. Combinations of microangiopathic/prediabetic neuropathy with inflammatory or toxic neuropathy are frequent especially in older patients. Hereditary neuropathy with concomitant neuritis is another important issue in this context [64, 65, 66]. 

## Conclusions and outlook 

In conclusion, nerve biopsy provides valuable information that contributes to the classification and differential diagnosis of neuropathies and helps to characterize their extent and course. Nerve biopsy is particularly important and useful for the diagnosis of non-hereditary, in particular inflammatory neuropathies. Seemingly sporadic neuropathies may be assigned to a hereditary disorder, and in cases of ambiguous molecular genetic findings, a nerve biopsy can provide clues towards the pathogenic gene mutation or at least highlight a possible cause and mechanisms for the neuropathy. With the advent of next generation sequencing, ambiguous genetic testing results are expected to become more frequent. Nerve biopsy analysis including studies on RNA and protein extracted from the tissue may become more useful to determine which gene alterations or combinations thereof caused the phenotype. Finally, scientific nerve biopsy analysis has contributed greatly to our understanding of peripheral neuropathies. Combined with new molecular genetic and cell biology methods and in conjunction with the examination of the ever growing number of animal models it will continue to contribute informative findings in the future. 

## Acknowledgments 

This study was supported in part by the German Research Council (DFG; WE 1406/13-1) and the IZKF Aachen (N5-3). We thank Mrs. L. Eshuis, Mrs. A. Knischewski, Mrs. H. Wiederholt, Mrs. C. Krude, Mrs. E. Beck and Mrs. H. Mader for technical support. 

**Table 1.** Features to be described in any nerve biopsy report.

Status of the epineurium including blood vessels
Alterations of the perineurium (thickening, fibrosis, calcification)
Endoneurial edema
Density of large and small myelinated nerve fibers
Extent of axonal degeneration and atrophy
Frequency of bands of Büngner and macrophages containing myelin debris
Number of macrophage clusters (CD68 staining)
Regeneration clusters
Demyelinated/remyelinated fibers
Onion bulb formations
Inflammatory infiltrates
Presence/absence of amyloid

**Figure 3.**
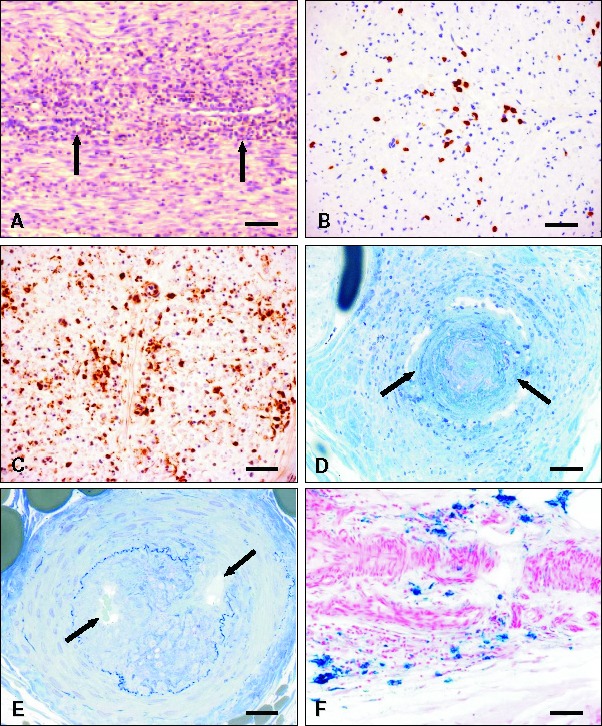
A: Endoneurial infiltration by numerous mononuclear inflammatory cells (arrows) in Guillain-Barré syndrome (GBS). H&E. Scale bar = 80 µm. B: Endoneurial cluster of CD8 immunoreactive cytotoxic T cells in chronic inflammatory demyelinating neuropathy, CIDP. Scale bar = 70 µm. C: Endoneurial clusters of CD68 immunoreactive macrophages in CIDP. Scale bar = 70 µm. D: Acute inflammatory infiltration of a small epineurial artery associated with fibrinoid necrosis (arrows) in vasculitic neuropathy. Semithin section, toluidine blue. Scale bar = 50 µm. E: Residual state after inflammatory lesion and occlusion of a small epineurial artery. The elastic lamina is irregular and focally interrupted. Arrows: small recanalizing vessels. Semithin section, toluidine blue. Scale bar = 40 µm. F: Perivascular epineurial hemosiderin deposits (blue) in chronic vasculitis. Turnbull blue. Scale bar = 100 µm.

**Figure 4.**
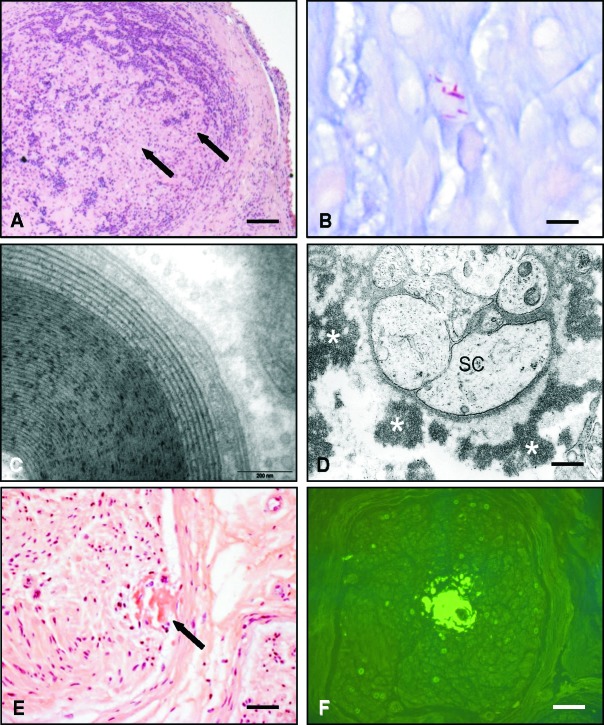
A: Tuberculoid leprosy: replacement of endoneurial structures by chronic lymphocytic infiltrates and granulomata (arrows). H&E. Scale bar = 120 µm. B: Acid-fast bacilli (red) in lepromatous leprosy. Wade-Fite stain. Scale bar = 25 µm. C: Widening of the spaces between the external myelin lamellae in monoclonal gammopathy. Immunohistochemistry had proven that these lesions were caused by IGG deposition in this case. EM. D: Endoneurial extracellular IGM deposits (asterisks) in a case of polyneuropathy associated with Waldenström’s disease. The specificity has been controlled by immuno EM. SC = Schwann cell. EM. Scale bar = 0.5 µm. E: Subperineurial amyloid deposits stained with Congo red (arrow) in amyloid neuropathy. Scale bar = 50 µm. F: Another endoneurial amyloid deposit in the same case, now stained with thioflavin S (bright green). Scale bar = 60 µm.

**Figure 5.**
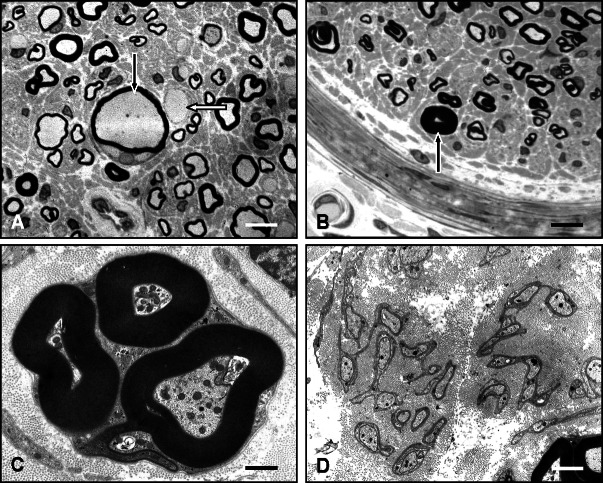
A: Enlarged myelinated (black arrow) and unmyelinated (white arrow) axons in giant axon neuropathy. Semithin section, toluidine blue. Scale bar = 15 µm. B: Tomaculous fiber (arrow) in hereditary neuropathy with liability to pressure palsy (HNPP). Semithin section, toluidine blue. Scale bar = 20 µm. C: Excessive myelin outfoldings in a case of CMT4C. EM. Scale bar = 2 µm. D: Abnormal connections and branching of Schwann cell processes of unmyelinated axons in another case of CMT4C. EM. Scale bar = 2 µm.

**Figure 6.**
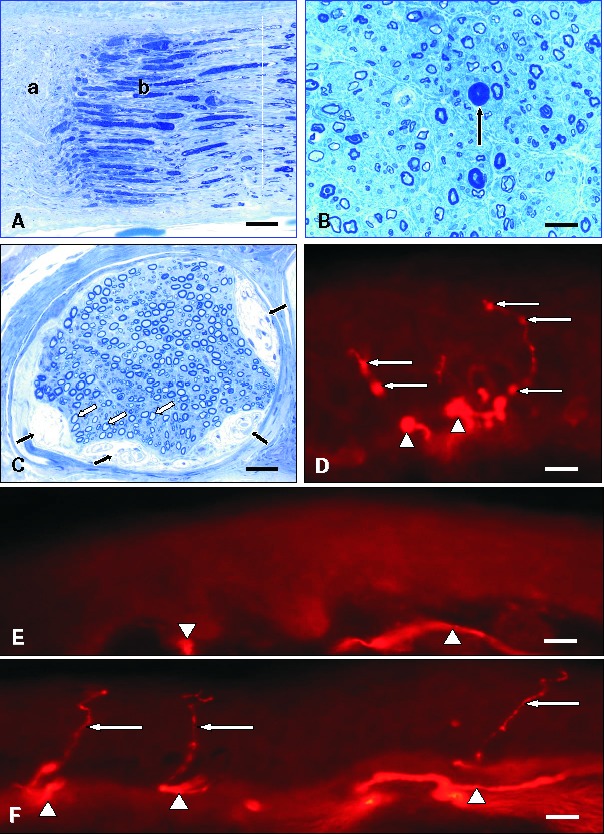
A: “Toothpaste” artefact. Compression of the nerve biopsy specimen leads to removal of myelin in sector “a”, mimicking myelinated nerve fiber loss, and “hypermyelination” in sector “b”. Dotted white line: level of the cross section depicted in B. Semithin section, toluidine blue. Scale bar = 40 µm. B: Cross section of a nerve fascicle that was crushed during surgical removal. The cross section is obtained from an area corresponding to the dotted line in A. Arrow: “Pseudo-tomaculous” fiber showing artificial hypermyelination. Semithin section, toluidine blue. Scale bar = 25 µm. C: Nerve fascicle containing several subperineurial Renaut bodies (black arrows). Note that many large nerve fibers especially in the vicinity of the Renaut bodies show disproportionately thin myelin sheaths most likely due to chronic compression. Semithin section, toluidine blue. Scale bar = 40 µm. D: Skin punch biopsy section from a patient with neuropathy. PGP 9.5 and Cy3 immunohistochemistry. Note bead-like enlargements along the intraepidermal nerve fibers (arrows) and focal swellings (arrowheads). Scale bar = 15 µm. E: Complete loss of epidermal nerve fibers in a skin punch biopsy of a patient with neuropathy. Remnants of the subepidermal nerve plexus are marked by arrowheads. PGP 9.5 and Cy3 immunohistochemistry. Scale bar = 20 µm. F: Normal skin innervation. The fascicles of the subepidermal nerve plexus (arrowheads) give rise to intraepidermal nerve fibers (arrows). PGP 9.5 and Cy3 immunohistochemistry. Scale bar = 30 µm.
